# Deep Convolutional Neural Support Vector Machines for the Classification of Basal Cell Carcinoma Hyperspectral Signatures

**DOI:** 10.3390/jcm11092315

**Published:** 2022-04-21

**Authors:** Lloyd A. Courtenay, Diego González-Aguilera, Susana Lagüela, Susana Del Pozo, Camilo Ruiz, Innes Barbero-García, Concepción Román-Curto, Javier Cañueto, Carlos Santos-Durán, María Esther Cardeñoso-Álvarez, Mónica Roncero-Riesco, David Hernández-López, Diego Guerrero-Sevilla, Pablo Rodríguez-Gonzalvez

**Affiliations:** 1 Department of Cartographic and Terrain Engineering, Higher Polytechnic School of Ávila, University of Salamanca, Hornos Caleros 50, 05003 Ávila, Spain; daguilera@usal.es (D.G.-A.); sulaguela@usal.es (S.L.); s.p.aguilera@usal.es (S.D.P.); ines.barbero@usal.es (I.B.-G.); 2 Department of Didactics of Mathematics and Experimental Sciences, Faculty of Education, Paseo de Canaleja 169, 37008 Salamanca, Spain; camilo@usal.es; 3 Department of Dermatology, University Hospital of Spain, Paseo de San Vicente 58-182, 37007 Salamanca, Spain; croman@saludcastillayleon.es (C.R.-C.); javier.canueto@gmail.com (J.C.); jcsantosduran@hotmail.com (C.S.-D.); mesthercardenoso@gmail.com (M.E.C.-Á.); monironcero@hotmail.com (M.R.-R.); 4 Instituto de Investigación Biomédica de Salamanca (IBSAL), Paseo de San Vicente 58-182, 37007 Salamanca, Spain; 5 Institute of Molecular Biology and Cellular Cancer (IBMCC), Centre of Cancer Investigation (Lab 7), Campus Miguel de Unamuno s/n, 37007 Salamanca, Spain; 6 Institute for Regional Development, University of Castilla la Mancha, Campus Universitario s/n, 02071 Albacete, Spain; david.hernandez@uclm.es (D.H.-L.); dguerrero@usal.es (D.G.-S.); 7 Department of Mining Technology, Topography and Structures, University of León, Av. Astorga s/n, 24401 Ponferrada, Spain; p.rodriguez@unileon.es

**Keywords:** basal cell carcinoma, hyperspectral sensor, computational learning, convolutional neural networks, support vector machines

## Abstract

Non-melanoma skin cancer, and basal cell carcinoma in particular, is one of the most common types of cancer. Although this type of malignancy has lower metastatic rates than other types of skin cancer, its locally destructive nature and the advantages of its timely treatment make early detection vital. The combination of multispectral imaging and artificial intelligence has arisen as a powerful tool for the detection and classification of skin cancer in a non-invasive manner. The present study uses hyperspectral images to discern between healthy and basal cell carcinoma hyperspectral signatures. Upon the combined use of convolutional neural networks, with a final support vector machine activation layer, the present study reaches up to 90% accuracy, with an area under the receiver operating characteristic curve being calculated at 0.9 as well. While the results are promising, future research should build upon a dataset with a larger number of patients.

## 1. Introduction

Skin cancer, including melanoma and non-melanoma, is one of the most common types of cancer, especially among white-skinned and elderly populations, with incidence rates still on the rise [1,2,3,4]. Although cases of non-melanoma skin cancer (NMSC) present a lower mortality rate than melanoma, its incidence rate is up to 20 times higher. The most common types of NMSC are basal cell carcinoma (BCC) and cutaneous squamous cell carcinoma (SCC or cSCC). BCC represents the majority of cases, while the steady increase in BCC patients suggests that this type of cancer may prevail over other types of skin cancer combined [3].

Skin malignancies are firstly evaluated by visual screening. For suspicious lesions, this evaluation is usually followed by biopsy and histopathological analyses of skin tissues. The high importance of early diagnosis has encouraged the development of methodologies for the automatic and more efficient detection of these skin lesions. This process is a challenging task, however, due to the large variability of skin lesions. Nevertheless, computational learning (CL) strategies, such as machine and deep learning, have emerged as powerful tools for these sorts of classification problems [5].

Several studies focus on the capacities of CL for skin cancer detection and classification [5,6]. In spite of this, not all pathologies are covered according to their importance and prevalence (Table 1). While recent years have seen an increase in the number of publications focusing on NMSCs, cancer types such as BCC, which are by far the most common [7], have received relatively little attention in the field of artificial intelligence (AI). Early detection of BCC is important considering the locally destructive nature of the disease and its increased depth of invasion, as well as difficulties in treatment for more severe cases [8,9]. Moreover, BCCs can present a possible increase in metastasis rates, with an increase of 1–2% if treatment is delayed for tumors >3 cm in diameter and an increase of up to 50% for tumors <10 cm [10]. From this perspective, the development of tools for tumor detection and analysis may speed up clinical practice by presenting a new means of planning treatments, and interventions, as well as studying lesions.

Computer vision has proven to have great potential for the non-invasive detection and classification of skin cancer lesions. While some methodologies have proven useful using conventional cameras in the visible range of the electromagnetic spectrum [5,11,12], hyperspectral imagery is also widely used [13]. Hyperspectral cameras have a high number of bands or channels, usually with wavelengths beyond the visible areas of the spectrum. Such sensors provide a large quantity of information that can be used for tumor identification and classification [14,15]. Nevertheless, hyperspectral images present the distinct disadvantage of being very large and hard to process. From this perspective, a very large dataset would be required for the efficient processing of these images using most CL techniques.

A recent study analyzed the spectral information provided by near-infrared hyperspectral images [16]. Using a pixel-based approach, said study employed robust statistical tests for feature selection. The authors were able to identify an optimal electromagnetic window that can be used to distinguish between different types of NMSC, as well as healthy skin. The present study develops this, employing this pixel-based approach to find an optimal neural network architecture for the classification of hyperspectral signatures.

In the field of applied CL in computer vision, convolutional neural networks (CNNs) can be considered a state-of-the-art algorithm for most image processing applications [17]. Part of this success is due to the wide-spread research into optimal CNN architectures, including ImageNet [18], VGG [19], ResNet [20], and Inception [21]. In light of this, CNNs have also been found useful for the processing of vectorial data [22], such as raw audio-waveforms [23], seismic vibrations [24], and electrocardiogram data [25]. CNNs can thus be considered a versatile set of algorithms, being adept at the extraction of spatially or temporally invariant features while reaching high performance in competition with, or even surpassing, human specialists [17].

Other algorithms such as support vector machines (SVMs) have also proved highly efficient for the processing of complex data types. Useful for both regression and discriminant analyses, SVMs use a *kernel trick* to overcome traditional limitations imposed by data linearity and normality [26]. From this perspective, SVMs have proven highly useful for the processing of different types of information, including some applications in computer vision [13,27], natural language processing [28,29], material quality inspection [30], and geometric morphometrics [31].

The concept of a neural support vector machine (NSVM) was originally proposed as a means of adding “depth” to SVMs [32], while using non-linear neural network architectures as a means of training a specified kernel function directly on the dataset. The added use of neural networks thus adds a highly versatile and flexible “kernel” for the SVM. Applications inspired by this approach have found NSVMs to be highly useful for both regression and classification tasks [33], extracting high-level features from low-level domains [34]. NSVMs have since proven successful in the processing of geometric data derived from 3D models [31], as well as other promising approaches in the classification of hyperspectral images [35].

The present study builds on each of the predefined approaches, using a convolutional NSVM (CNSVM) for the classification of BCC hyperspectral signatures. The present architecture employs 1D inception modules for convolution over hyperspectral data, performing feature extraction and thus acting as a kernel for the SVM activation layer. Both the network and the final SVM activation layer are fine-tuned using Bayesian approaches, reaching up to 90% overall accuracy in differentiating between healthy and cancerous tissue. Upon defining this architecture, future research including larger sample sizes may be a strong starting point for the classification and segmentation of entire hyperspectral images, facilitating diagnosis, patient screening, and the delimitation of cancerous regions.

## 2. Materials and Methods

### 2.1. Sample

**Figure 1:**
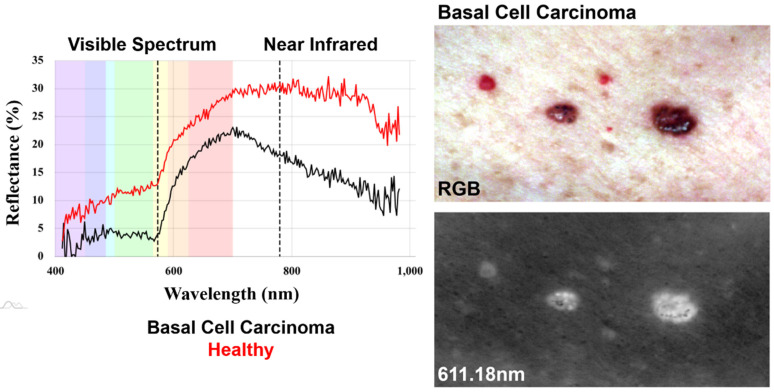
Examples of the hyperspectral signatures and images of healthy skin and basal cell carcinoma tumors.

The data set used in the present study was derived from previous works by Courtenay et al. [16], available online from [36]. This dataset consists of a total of 1505 hyperspectral signatures of three different samples, including BCC, cSCC, and healthy (H) skin samples (Figure 1).

Hyperspectral signatures for each patient were obtained using a Headwall Nano-Hyperspec visible–near-infrared (VNIR) hyperspectral imaging sensor. This particular sensor is a pushbroom linear camera, producing a vectorial array of pixels (1 × 640 px), registering wavelengths between 398.08 and 995.20 nm with a 2.2 nm spectral bandwidth separation between channels. The sensor was fit onto an ad hoc platform controlled by an electronic module device, designed for the synchronization of the platform’s movement, illumination, and the sensor’s shutter speed. Calibration of the sensor was performed using a marker board and frame presenting a known reflectance pattern (Spectralon). Pixel values were then calculated and radiometrically corrected through this calibration process so as to produce reflectance values (in%) for each channel of each pixel. Final images thus consisted of a 431 × 851 × 260 (rows, columns, and channels) tensor (Figure 1), having ≈95.4 million numeric values and occupying ≈0.3 GB of memory. For more details about the image acquisition process, consult the work of Courtenay et al. [16].

For the purpose of processing and characterizing hyperspectral signatures, Courtenay et al. [16] defined regions of interest (ROIs) for each of the images, randomly sampling pixels from ROIs directly over the tumor, while H samples were extracted at ROIs furthest away from the tumor so as to avoid possible contamination. A total of 41 BCC patients were originally studied. While the dataset includes data from cSCC patients, these signatures originate from a much smaller number of patients and were thus excluded in the present study. In light of this, the final sample size included here consists of 504 BCC signatures and 488 H signatures, amounting to a total sample size of 992. Considering the presence of only two labels (BCC and H), the training of all deep learning algorithms was thus conceptualized as a supervised binary classification problem, discerning whether the hyperspectral signatures represented cancer (BCC = 1) or not (H = 0).

All patients agreed to participate in the study; however, due to patient anonymity and data protection, no further details have been disclosed.

### 2.2. Base Convolutional Neural Network Architecture

The present architecture takes as input hyperspectral signatures represented as an ℝ^n^ first-order tensor, where *n* represents the number of hyperspectral channels included for the characterization of each sample. The previous publication of this dataset employed robust statistical approaches to define an optimal window between 573.45 and 779.88 nm (Figure 1). This region was defined as the portion of the electromagnetic spectrum where statistical differences between samples are most likely to be present [16]. Under this premise, all hyperspectral signatures were cropped to only include wavelengths within this window, producing a final input of ℝ^94^.

**Figure 2:**
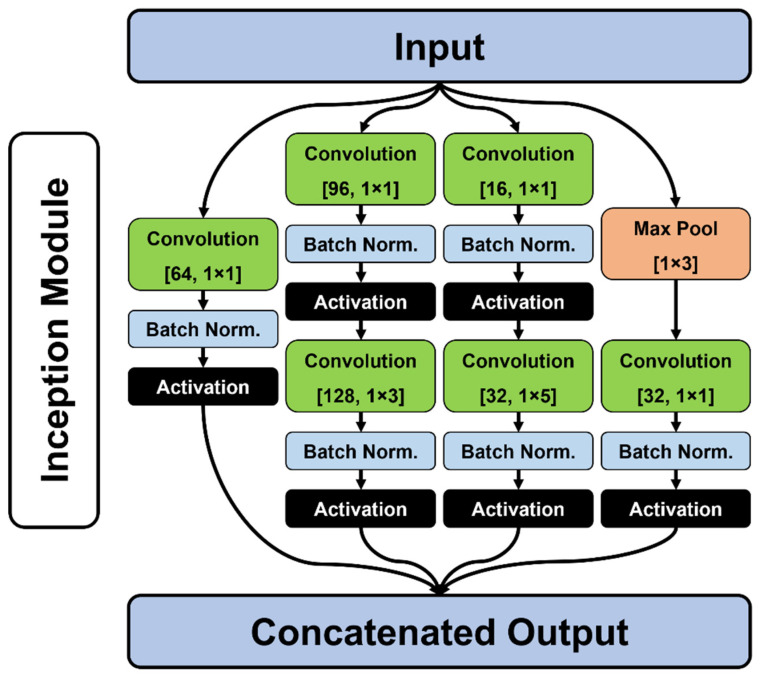
Figurative schematic representing the architecture of the 1D Inception modules used in the present study. Convolutional filters are described by [N° filters, receptive field (rows × columns)]. Batch Norm. indicates batch normalization, while activation layers depend on the configuration of the algorithm at the time of training.

The convolutional portion of the network is inspired by the Inception architecture, typically used in computer vision applications [21]. For this purpose, the inception module was adapted for 1D convolutions using a block of parallel convolutional layers of varying receptive field sizes (1 × 1, 1 × 3 or 1 × 5), as well as different numbers of filters per layer (Figure 2). Padding was used for all filters, while filter strides were set to 1 for all layers. Within the inception module, and parallel to these convolutional filters, an additional 3 × 1 max-pooling layer was also included. The output of the max-pooling layer was also passed into a convolutional filter (Figure 2).

**Figure 3:**
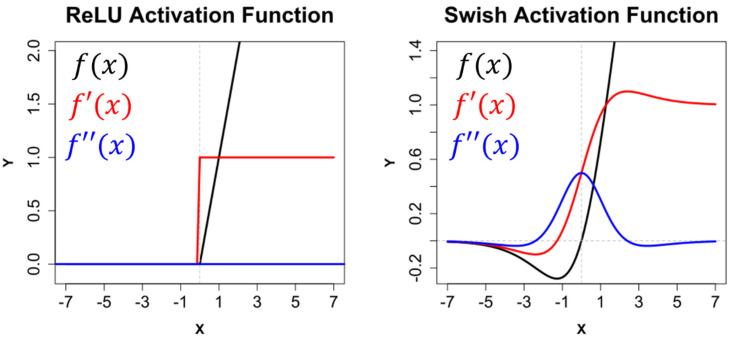
Graphical representation of the rectified linear unit (ReLU) and the self-gated rectified (Swish) activation functions (f(x)), alongside their first (f′(x)) and second (f″(x)) derivatives.

After each convolutional layer, batch normalization was used prior to activation, while two different non-linear activation functions were tried and tested (Equations (1) and (2), Figure 3). These were the rectified linear unit (ReLU) (Equation (1)), and the self-gated rectified activation function (Swish) (Equation (2)) [37];

(1) f(x)=max(0,x)

(2) $$f\left(x\right)=x\cdot \frac{1}{1+e^{-x}}$$

Additional hyperparameter configurations considered the use of kernel initializers and regularizers in each of the convolutional layers. The best results were obtained using the LeCun normal initializer [38,39], as well as an *ℓ2* regularizer with a coefficient of 0.0001 [40]. The results of each module were concatenated before being passed on to the next portion of the algorithm.

Experiments were performed by stacking a different number of inception modules on top of each other, as well as employing the use of only a single inception module prior to the fully connected neural networks that followed. While fully convolutional networks were also experimented with, considering their success in other applications [23], the present study found these architectures to considerably over- or under-fit on our training data.

Following the convolutional layers of our model, the concatenated output was flattened into a large vector and subsequently passed into a dense fully connected neural network. Different experiments considered the size and density of these fully connected layers, while a final aggressive reduction tactic was employed, ensuring each subsequent layer to be at least half the size of its predecessor. In between each of these layers, dropout algorithms were inserted for a more efficient training. Similarly to the convolutional portion of our algorithm, both ReLU and Swish activation functions were considered [37]. In the original configuration of the neural network, the final layer consisted of a single sigmoid activated neuron.

Training used a *train:validation* split of 70:30%, while training data were shuffled for each epoch. In these initial experiments, no data augmentation techniques were performed.

CNN models were trained using either a vanilla stochastic gradient descent (SGD) algorithm, or the Adam optimization algorithm [41]. Cyclic learning rates (CLRs) were employed [42], using the *Triangular2* function (base *α* = 0.0001, max *α* = 0.01, step size = 8). Models were run for 100 to 500 epochs until convergence, using batch sizes of 16, 32, 64, and 128. Finally, algorithms were implemented in the Python v.3.7.2 programming language, using the Tensorflow 2.1.0 library [43], and the TensorBoard toolkit for callback evaluation during training.

### 2.3. Convolutional Neural Support Vector Machines

Once the base architecture of the CNN had been trained, the final sigmoid activated layer was removed, replaced, and retrained with an SVM activation layer. For this purpose, each hyperspectral signature was passed through the CNN for feature extraction and then used to train the SVM [31]. For the tuning of this layer, *k*-fold cross-validation (*k* = 10) was used, while additional experiments alternated between a linear, polynomial, or radial SVM kernel functions.

The SVM portion of our network was implemented using the Scikit Learn v.0.22.1 library [44].

### 2.4. Hyperparameter Optimization

Hyperparameter optimization for multiple components of each model was performed using Bayesian optimization algorithms (BOAs) [45,46]. For the CNN component of our algorithms, BOAs were used to define targeted values such as the optimal dropout threshold, optimal parameters of *ℓ2* regularization, optimal neural layer densities, and optimal learning rates for CLR tuning. Similarly, for the SVM portion of our model, BOAs were used to define the optimal coefficients used in the kernel function. For all experiments, BOAs were run for 100 iterations, using an expected improvement selection function via the Tree of Parzen Estimator algorithm [46].

### 2.5. Model Evaluation

Six different performance indices were recorded for the evaluation of the present architecture, calculated on the test set for each of the experiments. The first of these included overall accuracy, sensitivity (i.e., true positive rate), and specificity (i.e., true negative rate) [47]. While other studies frequently use additional calculations such as precision and recall, these metrics are more suitable for studies presenting class imbalance [48]. Considering how this is not the case here, these metrics were thus excluded. Alongside specificity and sensitivity calculations, receiver operating characteristic (ROC) curves were also calculated, with their corresponding area under curve (AUC) values [49]. In addition to this, the kappa (*κ*) statistic was also used to assess the probability of agreement between the predicted output and the original label [47,50,51]. Finally, a calculation of the model’s final loss was performed using the mean squared error (MSE) metric.

For the evaluation of these metrics, a model was considered powerful if specificity and sensitivity values appeared balanced (sensitivity ≈ specificity), thus implicating high AUC values (<0.8). For kappa statistics, κ < 0.8 was considered as a threshold for almost perfect agreement between the real and predicted labels [47,50,51]. Finally, overall accuracy was only considered a reliable metric if all of these criteria had been met. 

### 2.6. Computational Facilities

For experimental purposes, three different computer systems were used for training, taking note of training time, CPU efficiency, and RAM usage. This was performed to assess not only the computational cost of the algorithms, but also their replicability on any standard computer system.

The first of these systems was a Dell Precision T1700 desktop computer, equipped with an Intel Xeon E3-1240 v3 CPU processor (4 cores, 3.40 GHz operating frequency) and 8 GB of RAM. The second system was a portable ASUS X550VX portable laptop, equipped with an Intel Core i5-6300HQ CPU processor (4 cores, 2.30 GHz) and 8 GB of RAM. Finally, the services provided by the Supercomputation Center of Castilla y Leon (SCAYLE) were also experimented with, employing the use of the Broadwell architecture for CL. This SCAYLE server has 2 Intel Xeon E5-2695 v4 CPU processors (18 cores each, 2.10 GHz), and 384 GB of RAM. In addition to this, this SCAYLE server provides access to a total of 8 NVidia Tesla v100 GPUs. For this purpose, experiments were performed using different numbers of CPU cores, as well as the use of GPUs, for training and data processing.

## 3. Results

**Figure 4:**
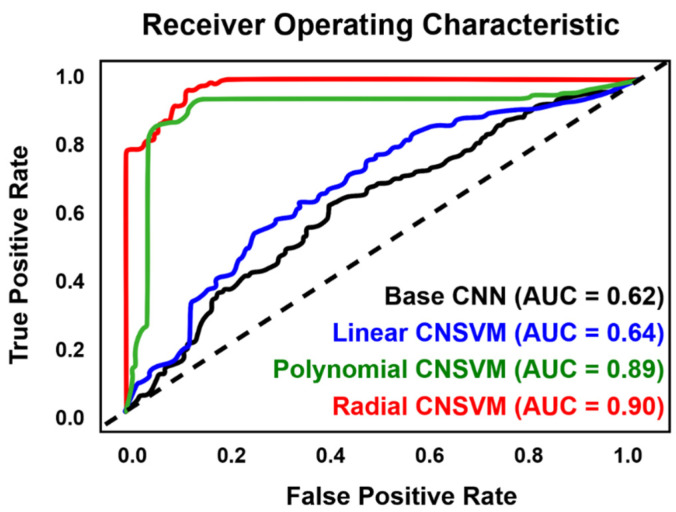
Receiver operating characteristic curves alongside their calculated area under curve (AUC) statistics for the different support vector machine activations used. CNN = base convolutional neural network without support vector machine activation. CNSVM = convolutional support vector machine.

Of all the configurations tried and tested, the model architecture that was found to produce the best results consisted of two 1D inception modules, feeding into a four-layer neural network, with densities of 200, 150, 100, and 50 neurons respectively (Table 2). This resulted in a final model of ≈5 million trainable parameters. Across all cases, models were found to converge after 400 epochs, while batch sizes of 128 provided algorithms with enough information to successfully train from. CNN architectures by themselves were not reported to produce promising results for the classification of hyperspectral signatures, reaching an accuracy of only approximately 56.7%, AUC of 0.61, and *κ* values as low as 0.3. Nevertheless, with the inclusion of the final SVM activation layer, CNSVM algorithms presented between a 12.1% and a 49.3% improvement in performance (Figure 4), with the radial SVM algorithm reaching the highest recorded results (accuracy = 91.0%, *κ* = 0.81, AUC = 0.91). Table 2 presents the final CNSVM architecture used in this study.

**Figure 5:**
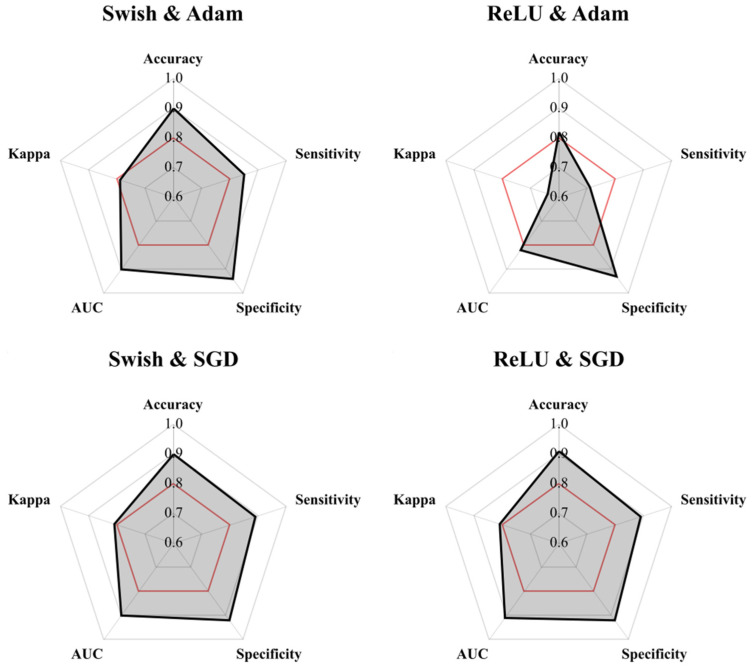
Radar plots comparing performance metrics of each of the configurations tried and tested. AUC = area under curve. ReLU = rectified linear unit. SGD = stochastic gradient descent. The red line at 0.8 marks a suitable threshold defining an optimal computational learning model.

When fine-tuning the CNSVM considering the different types of activation functions and optimization algorithms, both Swish and ReLU were observed to produce optimal results (Table 3, Figure 5). Nevertheless, Adam was observed to fit the Swish models better than ReLU, while SGD produced the best results on all accounts (Table 3, Figure 5). Finally, MSE results reveal all models to produce confident predictions, with the Swish and SGD algorithm reaching the best results, as seen by a mean of 97.1% confidence with each prediction made on the test set.

Finally, when considering the computational cost of these algorithms, as would be expected, training time was considerably conditioned by the inclusion of a GPU (Table 4), while the higher number of CPU cores also reduced training times. The longest recorded training time was registered at 40 min when using the personal laptop (Table 4), with each epoch taking 5.9 s. The inclusion of a single GPU was found to train neural networks 24 times faster. The time taken to tune the SVM layer via BOAs was not seen to be affected by the number of CPUs, with all computer systems taking ≈1.7 min to tune the final activation layer and 0.01 min to fit. As for computational resources, all computers required ≈1.5 GB of RAM for the training of models, while CPU efficiency was recorded at 80%. In sum, the CNSVM for hyperspectral signature classification can easily be trained without the need for high computational facilities.

## 4. Discussion

The present study proposes a convolutional neural support vector machine architecture for the classification of healthy skin and basal cell carcinoma hyperspectral signatures. As can be seen, the network works well on test sets, reaching up to 90% classification in terms of overall accuracy, producing highly confident predictions, and is relatively easy to train. Multiple experiments throughout this paper have found both the Swish and ReLU activation functions to perform well, with ReLU performing marginally better in some applications. Finally, the results presented here can be considered an additional example of how support vector machine activation layers can prove highly effective when combined with deep neural network architectures.

**Figure 6:**
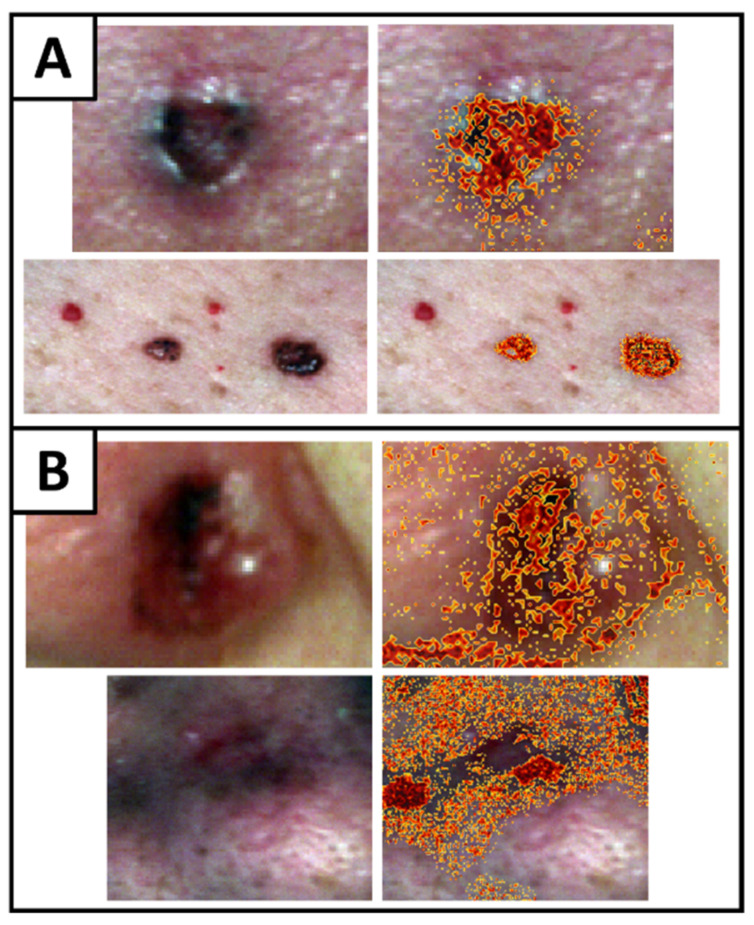
Preliminary examples of (**A**) good and (**B**) poor image segmentation using CNSVMs for the classification of each pixel. (**A**) Examples of BCC tumors found on the forehead of a male patient and shoulder of a female patient. (**B**) Examples of BCC tumors found in the crease between the cheek and nostril of two female patients. Due to patient anonymity, images have been cropped to avoid revealing any distinguishing features.

Preliminary use of the CNSVM model to classify every pixel in an image proved quite successful (Figure 6A). Nevertheless, some issues were found in images presenting poorer lighting conditions (Figure 6B). In these latter cases, it can be seen how areas of shadow that are typically found in the creases and wrinkles of many patients’ faces evidently reflect less light. This feature unfortunately is what predominantly separates BCC from healthy skin [16]. Similarly, preliminary observations noted how BCC presents a large inter-patient variability, implying that a larger sample of cancer patients should definitely be considered in the future.

While each of these limitations are noteworthy, these can be overcome through the collection of larger datasets that will better represent the variability of the lesions, as well as the incorporation of improvements to the lighting setup of the sensor’s platform (as noted by [16]). Moreover, CNSVMs still proved to be highly successful when learning from this data, showing how future applications employing transfer learning [52,53], or developed generative data augmentation techniques [31,54,55,56], are still likely to perform well. Each of these steps must be considered fundamental before applications in real clinical settings.

The goal of this study was to develop previous observations regarding the differences observed via robust statistical techniques [16]. Here we have shown how advanced computational techniques are able to effectively learn these differences, precisely in the spectral range between 573.45 and 779.88 nm. Linked with other research in the field of hyperspectral imagery and dermatological analyses, other regions of the electromagnetic spectrum may be considered useful, branching out further into the near-infrared (1000 to 1700 nm) or short-wave infrared (1000 to 2500 nm) portion of the spectrum. This is especially relevant for the study of some types of skin cancer, such as melanoma [13] and cSCC [57].

Needless to say, CNSVMs can be considered a valuable tool for the processing of this type of data, presenting an important framework to build upon for more developed and advanced applications in real clinical settings.

## Figures and Tables

**Table 1:** A bibliographical summary of the number of scientific publications registered in the arXiv (https://arxiv.org/) and Science Direct (https://www.sciencedirect.com/) databases presenting the terms “machine learning” (ML) and “deep learning” (DL) in relation with different types of skin cancer (consulted 1 July 2021). Searches considered the appearance of these terms in either the abstract, title, or keywords.

	arXiv		Science Direct	
	ML	DL	ML	DL
Skin Cancer	47	56	43	71
Non-Melanoma Skin Cancer (NMSC)	2	3	5	5
Melanoma	13	15	75	78
Cutaneous Squamous Cell Carcinoma (SCC/cSCC)	3	3	5	2
Basal Cell Carcinoma (BCC)	6	3	5	9

**Table 2:** Description of the final model architecture used for the supervised classification of hyperspectral signatures. The 1D Inception module blocks are constructed following the architecture presented in Figure 2.

Convolutional Neural Support Vector Machine	
Input: 1 × 94 Vector Hyperspectral Signature	
1D Inception Module	
Concatenation	
1D Inception Module	
Concatenation	
Flattening	
Dropout	*p* = 0.54
Dense	*nº* = 200
Dropout	*p* = 0.33
Dense	*nº* = 150
Dropout	*p* = 0.10
Dense	*nº* = 100
Dropout	*p* = 0.46
Dense	*nº* = 50
Radial Kernel Support Vector Machine Activation	
*Binary Output label: Healthy (0) or BCC (1)*	

**Table 3:** Algorithm performance on test sets. AUC = area under curve. MSE = mean squared error. ReLU = rectified linear unit. SGD = stochastic gradient descent.

	Swish and Adam	ReLU and Adam	Swish and SGD	ReLU and SGD
Accuracy	0.90	0.82	0.90	0.91
Sensitivity	0.85	0.71	0.89	0.89
Specificity	0.94	0.93	0.92	0.92
AUC	0.90	0.82	0.90	0.91
Kappa	0.79	0.64	0.81	0.81
MSE	0.034	0.078	0.029	0.035

**Table 4:** Model training time (seconds per epoch) using different computer systems as well as specifying the number of CPUs and GPUs made available to Tensorflow during training.

Computer	No. CPUs	No. GPUs	Seconds/Epoch
Personal Laptop	4	0	5.94
Desktop Computer	4	0	4.75
SCAYLE	4	0	5.36
SCAYLE	10	0	2.68
SCAYLE	18	0	1.86
SCAYLE	4	1	0.25
SCAYLE	18	1	0.20

## Data Availability

All data used in the current study are located at the corresponding author’s GitHub repository: https://github.com/LACourtenay/HyperSkinCare_Statistics (accessed on 2 September 2021).

## Code Availability

All code used in the current study is located at the corresponding author’s GitHub repository: https://github.com/LACourtenay/HyperSkinCare_DL (accessed on 2 September 2021).
